# A Single-Batch Fermentation System to Simulate Human Colonic Microbiota for High-Throughput Evaluation of Prebiotics

**DOI:** 10.1371/journal.pone.0160533

**Published:** 2016-08-02

**Authors:** Risa Takagi, Kengo Sasaki, Daisuke Sasaki, Itsuko Fukuda, Kosei Tanaka, Ken-ichi Yoshida, Akihiko Kondo, Ro Osawa

**Affiliations:** 1 Department of Bioresource Science, Graduate School of Agricultural Science, Kobe University, 1–1 Rokkodai-cho, Nada-ku, Kobe, Hyogo 657–8501, Japan; 2 Graduate School of Science, Technology and Innovation, Kobe University, 1–1 Rokkodai-cho, Nada-ku, Kobe, Hyogo 657–8501, Japan; 3 Organization of Advanced Science and Technology, Kobe University, 1–1 Rokkodai-cho, Nada-ku, Kobe, Hyogo 657–8501, Japan; 4 RIKEN Center for Sustainable Resource Science, 1-7-22 Suehiro-cho, Tsurumi-ku, Yokohama, Kanagawa 230–0045, Japan; 5 Research Center for Food Safety and Security, Graduate School of Agricultural Science, Kobe University, 1–1 Rokkodai-cho, Nada-ku, Kobe, Hyogo 657–8501, Japan; University of Illinois at Urbana-Champaign, UNITED STATES

## Abstract

We devised a single-batch fermentation system to simulate human colonic microbiota from fecal samples, enabling the complex mixture of microorganisms to achieve densities of up to 10^11^ cells/mL in 24 h. 16S rRNA gene sequence analysis of bacteria grown in the system revealed that representatives of the major phyla, including Bacteroidetes, Firmicutes, and Actinobacteria, as well as overall species diversity, were consistent with those of the original feces. On the earlier stages of fermentation (up to 9 h), trace mixtures of acetate, lactate, and succinate were detectable; on the later stages (after 24 h), larger amounts of acetate accumulated along with some of propionate and butyrate. These patterns were similar to those observed in the original feces. Thus, this system could serve as a simple model to simulate the diversity as well as the metabolism of human colonic microbiota. Supplementation of the system with several prebiotic oligosaccharides (including fructo-, galacto-, isomalto-, and xylo-oligosaccharides; lactulose; and lactosucrose) resulted in an increased population in genus *Bifidobacterium*, concomitant with significant increases in acetate production. The results suggested that this fermentation system may be useful for *in vitro*, pre-clinical evaluation of the effects of prebiotics prior to testing in humans.

## Introduction

The human gastrointestinal tract is colonized by a total number of ~10^14^ bacterial cells [1] of 400~1,000 species [2,3] to form a gut microbiota that has an important influence on the nutritional and health status of the host [4,5]. It has long been known that food components ingested by the hosts influence their gut microbiota, both quantitatively and qualitatively [4,6]. Consequently, various functional food components such as prebiotics, probiotics, and biogenics have attracted interest as potential ways to manipulate the gut microbiota and to improve human health [7,8]. Usually, the functionality of such food components has been evaluated by human intervention trials or animal-feeding trials [3,9]. However, human trials often are constrained by ethical considerations [10,11] while animal-feeding trials often give results that are not reproducible in humans, in part because of differences in microbiota composition between those of animals and humans [12,13]. Therefore, it was necessary to develop a relatively simple and short-term *in vitro* evaluation system that simulates the human colonic microbiota not only metagenomically (regarding the composition of microbiota) but also metabolically.

On the basis of the above considerations, many *in vitro* models simulating the human intestinal tract have been designed to evaluate the functionality or safety of food components [11,14–18]. Such *in vitro* models offer several advantages, including dynamic sampling over time and high reproducibility, without the ethical issues that can arise in clinical contexts. Previously, several dynamic, multi-compartment culture systems have been designed to replicate the mechanical characteristics of an entire and continuous gastrointestinal tract [10,19]; those systems usually required complex and longer-term experiments for evaluation of functional food components [17]. Selected molecular techniques, such as denaturing gradient gel electrophoresis and microarray analyses, have been used to perform phylogenetic analyses of the multi-compartment-culture intestinal models [17,20]. However, none of those models has been able to provide sufficient evidence to confirm that the mixture of microorganisms growing in the *in vitro* system truly represented the taxonomic diversity of the human microbiota [10,21].

Recent developments in molecular biological methods have permitted improved characterization of the gut microbial ecosystem [22]. Notably, next-generation sequencing (NGS) has facilitated the analysis of a large number of microorganisms in the human intestinal tract and has provided the reliable taxonomic information about the *in vivo* microbiota [1,23,24]. It may now be possible to analyze associations between the genes of the human intestinal microbiota and host health status. An extensive reference catalog of microbial genes present in the human intestine has been established [1]. In addition, bioinformatics for interpretation of the biological and genetic information is in development. These advances would permit the identification of person-to-person differences in microbial genes associated with disease, as well as the underlying mechanisms of host/microbe interactions [22].

However, the power of NGS has not yet (to our knowledge) been successfully applied to the comparison of the microbiota *in vivo* (in individual human colon) to those of *in vitro* model systems. Therefore aim of the present study was to construct a simple fermentation system, to provide a novel human colonic model that reliably simulated the composition of the *in vivo* microbiota as proved by NGS analysis. In addition, in order to substantiate the practicality of the model, we evaluated the functionality of prebiotics, in this case by testing oligosaccharides that are known to be capable of increasing numbers of genus *Bifidobacterium* in the human gut [8,25,26].

## Materials and Methods

### The single-batch fermentation system

Operations of the single-batch fermentation system were performed using a pH-controlled multi-channel fermenter (Bio Jr.8; ABLE, Tokyo, Japan) (S1 Fig). The simulator consisted of eight parallel and independent vessels. Fermentation was conducted at a 100-mL working volume per vessel. Anaerobic conditions of the vessels were maintained by purging with a mixture of N_2_ and CO_2_ (80:20) gas (15 mL/min) that was filter-sterilized through a 0.2-μm PTFE membrane (Pall Corporation, IL, USA) before the start of and during fermentation. Each fermentation was performed just once at 37°C with magnetic stirring at 300 rpm and continuous pH monitoring. To mimic the pH of the colon of a healthy adult, the pH was adjusted to 6.5 by addition of 1 M NaOH using an automatic pump of the fermenter system.

### Fecal inoculum and fermentation

Fecal samples were obtained from healthy human volunteers (n = 3 for bacterial evaluation using NGS analysis, and n = 6 for prebiotics evaluation using quantitative PCR analysis) who had not been treated with antibiotics for more than 3 weeks prior to sampling and were selected randomly from the youth to middle aged people. After collection, fecal samples were immediately placed under anaerobic conditions using AnaeroPack (Mitsubishi Gas Chemical Co., Inc., Tokyo, Japan). Each fecal sample was weighed and diluted 10-fold with 0.1 M phosphate buffer (0.1 M NaH_2_PO_4_: 0.1 M Na_2_HPO_4_ = 2:1; pH 6.5). Fermentation was initiated by inoculation of each medium-containing vessel with 100 μL of the fecal suspension (10% wt/vol). The human fecal samples were handled under the supervision of Takeshi Azuma, a licensed physician, in accordance with the guidelines of Kobe University Hospital, and a written informed consent was obtained from every volunteer. All the experimental protocols were approved by the institutional ethics review board at Kobe University. All the methods used in this study were in accordance with the approved guidelines by the Medical Ethics Committee at Kobe University. Aliquots of the fermentation cultures were sampled through the side projection of the vessel without disturbing the internal anaerobic conditions at 0, 6, 9, 12 and 24 h after the initiation of fermentation. Diluted feces and fermentation samples were stored at –20°C prior to the experiments.

### Medium and substrates

The medium in the single-batch fermentation system was based on Gifu anaerobic medium (GAM (Code 05422); Nissui Pharmaceutical Co, Tokyo, Japan) and was buffered to pH 6.5 by addition of phosphate buffer (0.1 M NaH_2_PO_4_: 0.1 M Na_2_HPO_4_ = 2:1; pH 6.5). The modified GAM was autoclaved at 115°C for 15 min before use. For evaluation of the prebiotics, one of the solutions of various oligosaccharides was added into one of the vessels to achieve a final concentration of 5 g/L, including fructooligosaccharide (FOS; Wako Pure Chemical Industries, Osaka, Japan), galactooligosaccharide (GOS; Wako), isomaltooligosaccharide (IMO; Wako), xylooligosaccharide (XOS; Wako), raffinose (Difco, BD, Le Pont de Claix, France), lactulose (Wako), or lactosucrose (LS-90P, Ensuiko Sugar Refining Co., Ltd., Tokyo, Japan). Each of the oligosaccharide solutions was sterilized by membrane filtration using Millex^®^ syringe filter units (pore size, 0.45 μm; Merck Millipore, Darmstadt, Germany) prior to using it. One of the vessels was assigned as the negative control without addition of oligosaccharide.

### Short-chain fatty acid analysis

Acetate, propionate, butyrate, lactate, and succinate were measured using a high performance liquid chromatograph (HPLC) (Shimadzu, Kyoto, Japan) equipped with an Aminex HPX-87H column (Bio-Rad Laboratories, Hercules, CA, USA) and RID-10A refractive index detector (Shimadzu) and operated at 65°C using 5 mM H_2_SO_4_ as the mobile phase at a flow rate of 0.6 mL/min.

### Isolation of bacterial DNA

Whole genomic DNA from each inoculum or culture was prepared according to the method of Marmur [27] with minor modifications. Briefly, a 200-μL aliquot of each culture was transferred to a sterilized and DNA-free bead-beating tube containing 300 mg of glass beads (diameter 0.1 mm). A volume of approximately 500 μL of TE(10 mM Tris-HCl, 1 mM EDTA, pH 8.0)-saturated phenol, 250 μL of lysis buffer, and 50 μL of 10% (w/v) sodium dodecyl sulfate were added to each tube. The mixture then was shaken vigorously for 30 s at 5.0 m/s in FastPrep-24 instrument (MP Biomedicals SARL, Illkirch, France). After centrifugation at 22,000 × g for 5 min, the upper layer was transferred to a fresh tube. DNA was extracted using 400 μL of phenol-chloroform-isoamyl alcohol (25:24:1) and the mixture was centrifuged at 22,000 × g for 5 min. The upper aqueous layer was transferred to another fresh tube containing 275 μL of isopropyl alcohol and a 1/10 volume of 3 M sodium acetate, and chilled at −20°C for 10–15 min. The extracted DNA precipitate was pelleted by centrifugation at 22,000 × g for 5 min, then washed with 70% ethanol and then dried under vacuum. The DNA subsequently was dissolved in TE.

### Illumina library generation

The V3–V4 region of the bacterial 16S rRNA gene was amplified using S-D-Bact-0341-b-S-17 (5’-CCTACGGGNGGCWGCAG-3’) and S-D-Bact-0785-a-A-21 (5’-GACTACHVGGGTATCTAATCC-3’) [28]. A 16S metagenomics sequencing library was prepared according to the manufacturer’s instructions (Illumina, San Diego, CA, USA). To normalize the sample amplicons, DNA concentrations in the PCR products were measured using the Qubit ds DNA HS Assay Kit (Thermo Fisher Scientific, MA, USA). An appropriate amount of the 16S rRNA gene products and an internal control (PhiX control V3; Illumina, Tokyo, Japan) was subjected to paired-end sequencing by a MiSeq sequencer (Illumina) with a 600-cycle MiSeq reagent kit (Illumina). 16S rRNA-targeted amplicon reads were taxonomically classified by the GreenGenes taxonomic database (Illumina) using the Ribosomal Database Project (RDP) Classifier as described by Wang et al. [29]. Shannon-Wiener index was calculated using the equation H = –∑[(pi)ln(pi)], where H is Shannon-Wiener index and pi is the proportion of total species represented by species i [30]. All the raw sequence data generated in this study have been deposited in MG-RAST as “Single Batch Fermentation System Simulating Human Intestinal Microbiota” under the accession numbers 4683354.3–4683365.3.

### Quantitative PCR analysis

Real-time quantitative PCR was performed with the TP700 Thermal Cycler Dice Real Time System Lite (Takara Bio, Ohtsu, Japan). The primer sets described in S1 Table were used for measuring the partial 16S rRNA gene copy number [31,32,33,34]. The quantitative measurement by real-time PCR was conducted in triplicate. The real-time PCR amplification program for eubacteria was as follows: 95°C for 3 min, followed by 38 cycles of 95°C for 30 sec and 54°C for 30 sec. The real-time PCR amplification program for the genus *Bifidobacterium* was as follows: 94°C for 5 min, followed by 40 cycles of 94°C for 20 sec, 55°C for 20 sec and 72°C for 50 sec. To check the specificity of the amplifications, a melting curve was obtained by performing the following cycle: a denaturation step at 95°C for 15 sec, a 1°C increase in temperature every 20 sec starting at 60°C and ending at 95°C, and a final step at 95°C for 15 sec. Standard curves for absolute quantification in the cultures were prepared using 10^2^–10^6^ copies of the PCR fragments of the 16S rRNA genes. The correlation coefficients for all the standard curves exceeded 0.99. For each assay, 2 μL of DNA solution was added to 18 μL of a PCR mixture containing 10 μL of THUNDERBIRD^™^ SYBR^®^ qPCR Mix (Toyobo, Osaka, Japan), 7.2 μL of distilled water, and 200 nM of each primer.

### Statistical analysis

Metagenomic data were normalized to the sum of the total genera numbers. A principal component analysis (PCA) of these normalized metagenomic data was performed using the JMP software package (version 12; Institute, Tokyo, Japan). Data from the negative control and the experimental samples with addition of each prebiotics were compared by the Dunnett test using the JMP software package (version 12). P-values below 0.05 were considered to be statistically significant.

## Results

### Operation of the single-batch fermentation system

The single-batch fermentation system was designed to simulate the human colon. Since more than 99% of the gut microbiota are obligate anaerobes [35], anaerobic conditions in our system were carefully constructed and maintained by using a previously defined medium for the growth of anaerobes, Gifu anaerobic medium (GAM) [36] with some modifications, and constantly flushing the medium with N_2_/CO_2_ gas before and during the fermentation. Operation of the batch fermentation system was initiated by inoculation with small amounts of one of the human volunteer fecal samples, which was designated as F37-1 (female, age 37, Japanese), F37-2 (female, age 37, Japanese), or M54 (male, age 54, Japanese). Within 24 h after the initiation of fermentation, the glucose originally contained in the medium was consumed completely, and microbial composition was not changed between 24 h and 30 h after the initiation of fermentation (S2 Table). Thus, the operational duration was set as 24 h in this study.

### Bacterial 16S rRNA gene sequence analysis on microbiota in human fecal samples and their corresponding single-batch fermentation cultures

**Fig 1:**
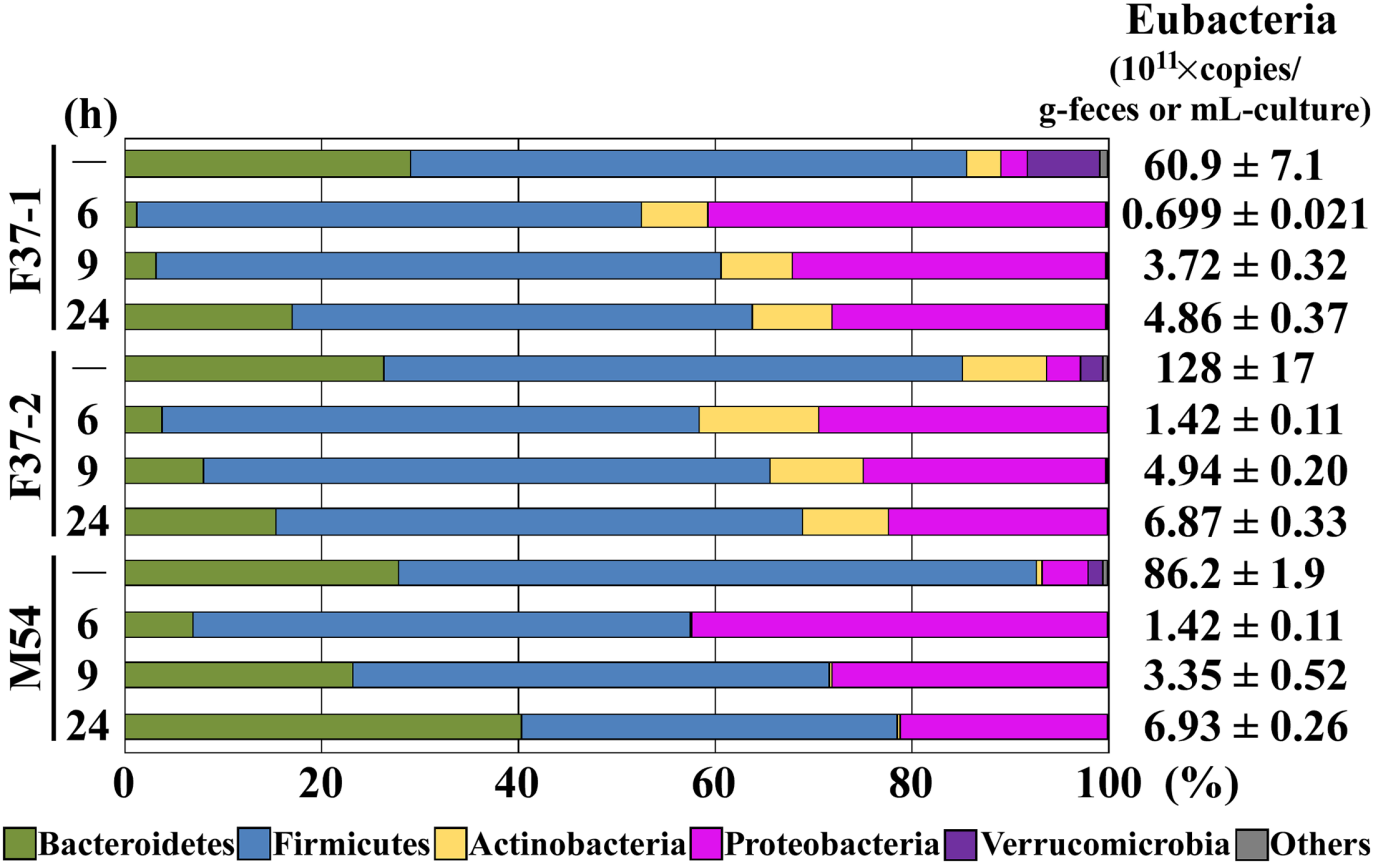
Phylum-level compositional view of bacteria in three human fecal samples/inocula (–) (F37-1: female, age 37; F37-2: female, age 37; and M54: male, age 54) and corresponding cultures at 6, 9, and 24 h after the initiation of fermentation. A compositional view of bacterial phyla based on the taxonomic assignment of 16S rRNA genes is shown. Bacterial composition of each sample was estimated from the results of the RDP classifier. Eubacterial 16S rRNA gene copy numbers are shown in the right column; units are copies/wet-g-feces (inoculum) or copies/mL-culture (fermentation).

The composition of representing human gut microbiota that developed in the single-batch fermentation system was examined in detail and compared with that of the original fecal samples. Total microbial DNA was isolated from each of the three fecal samples (F37-1, F37-2, and M54). Bacterial species are known to achieve the highest and representative cell densities among Bacteria, Archaea, and Eukarya contained in human fecal samples [37]. The total number of eubacteria was calculated to range from 60.9 × 10^11^ to 128 × 10^11^ copies/wet-g of feces. Bacterial 16S rRNA gene sequence analysis of the fecal samples was performed using NGS, yielding 12,283,776 quality reads with an average of 1,023,648 reads per sample. According to the obtained sequences, a total of 24 phyla and 524 genera were identified. Bacterial species belonging to phyla Bacteroidetes and Firmicutes have been reported to dominate human feces [1,2] and NGS analysis revealed that these two phyla represented 85.7, 85.2, and 92.6% of the total phyla identified in the fecal samples F37-1, F37-2, and M54, respectively (Fig 1). Species belonging to other phyla, Actinobacteria and Verrucomicrobia, were present in minor proportions but had been detected in the fecal samples used to inoculate the cultures. DNA samples from the fermentation cultures were isolated at 6, 9, and 24 h after inoculation into the batch system and then subjected to bacterial 16S rRNA gene sequence analysis. After 24 h of fermentation, eubacterial copy numbers reached 4.86 × 10^11^ to 6.93 × 10^11^ copies/mL (Fig 1). At the earlier stages of fermentation (6 and 9 h after inoculation), species belonging to phylum Bacteroidetes decreased in number, being replaced primarily by those of phylum Proteobacteria for cultures initiated with either F37-1, F37-2, or M54 inocula. Subsequently (at 24 h after the initiation of fermentation), the numbers of bacteria belonging to phylum Bacteroidetes gradually increased to replace those of Proteobacteria, achieving dominance, accounting for 63.8, 68.9, and 78.5% of the reads in the F37-1, F37-2, and M54 cultures, respectively. Proportions of species of phylum Actinobacteria were largely unchanged in the fermentation cultures, while those of phylum Verrucomicrobia drastically decreased as the fermentation proceeded.

The microbial composition was further examined at the genus level (S2 Fig). In all three cases, the majority of the species of phylum Bacteroidetes belonged to genus *Bacteroides* in both the original fecal samples and the single-batch fermentation cultures. In the inocula, bacteria of phylum Firmicutes consisted primarily of genus *Blautia*; this prevalence was maintained only in the M54 fermentation culture, while bacteria of genera *Enterococcus* and *Streptococcus*, which had been minor in the original F37-1 and F37-2 inocula, respectively, became the majority genera of this phylum in the corresponding fermentation cultures *in vitro*. Bacteria of the phylum Proteobacteria were primarily members of the genus *Escherichia*, both in the inocula and during culturing, and this pattern was largely extended in the fermentation cultures. In the fecal inocula, species of phylum Actinobacteria belonged mainly to genus *Bifidobacterium*, and were well maintained in the fermentation cultures. In contrast, the majority of the bacteria of phylum Verrucomicrobia in the original fecal samples belonged to genus *Akkermansia*, but most of these organisms were lost during the *in vitro* cultivation. Therefore, at the phylum level, the single-batch fermentation system maintained and simulated proportions of the majority of the bacterial species that dominated in the original fecal samples, including members of phyla Bacteroidetes and Firmicutes. However, at the genus level, the single-batch fermentation system did not always reproduce the microbial composition of the inoculating fecal samples.

**Fig 2:**
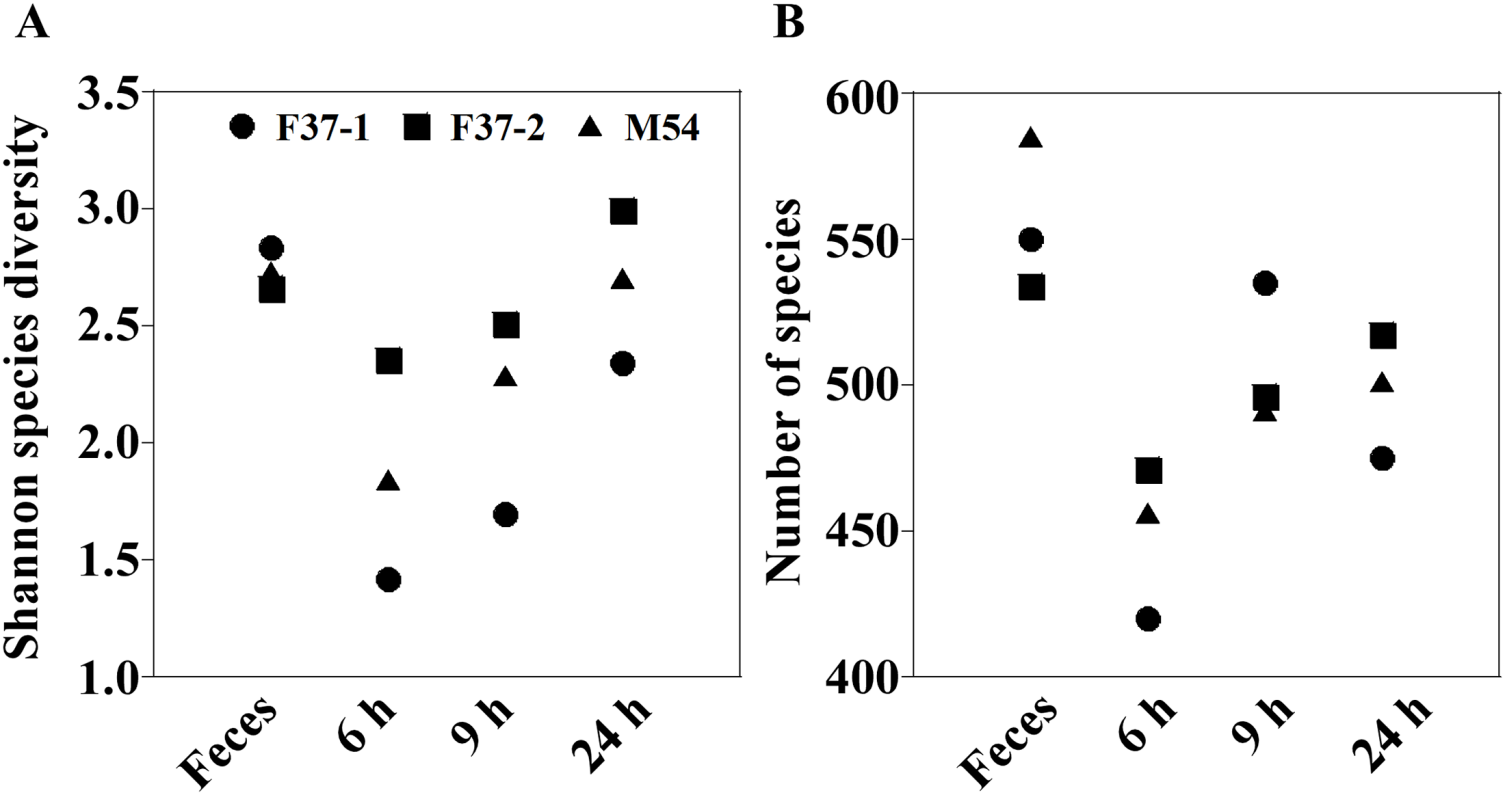
Bacterial diversity measures by observed species and Shannon-Wiener index in three human volunteers’ feces (F37-1: female, age 37; F37-2: female, age 37; and M54: male, age 54) and corresponding cultures at 6, 9, and 24 h after the initiation of fermentation. The Shannon-Wiener diversity index characterizes the diversity in a community, i.e., species abundance and evenness.

**Fig 3:**
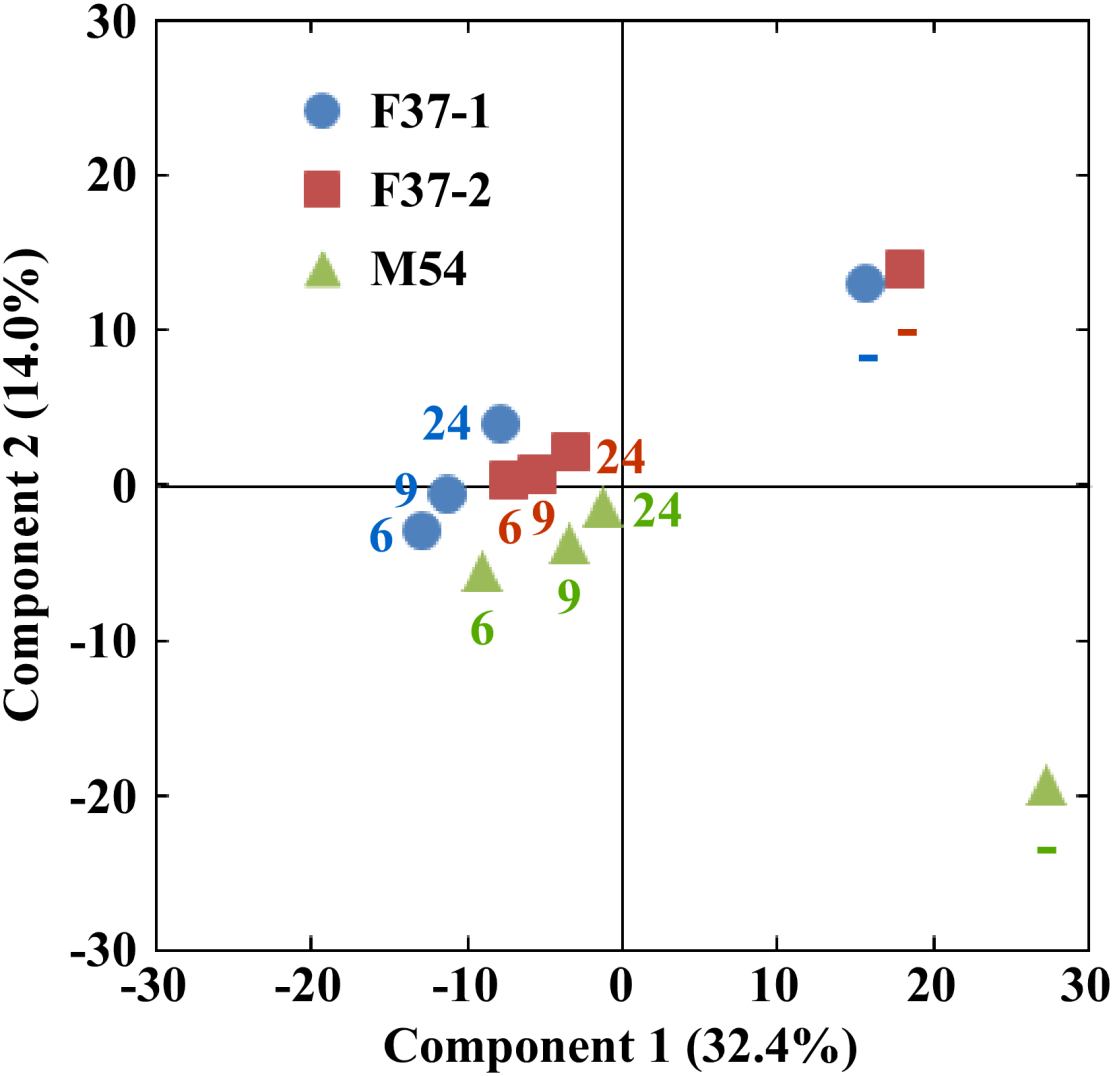
Principal component analysis (PCA) of 16S metagenomic data of bacterial species in three human volunteers’ feces (–) (F37-1: female, age 37; F37-2: female, age 37; and M54: male, age 54) and corresponding cultures at 6, 9, and 24 h after the initiation of fermentation. F37-1, F37-2, and M54 are indicated in blue, orange, and green, respectively. The numbers indicate the sampling time points.

The Shannon-Wiener index of the original human fecal samples (2.658–2.835) fell in fermentation cultures obtained at 6 h after the inoculation (1.418–2.350), but thereafter the index gradually increased up to the original levels by 24 h (2.340–2.996) (Fig 2A). Additionally, the numbers of species also exhibited changes similar to those seen by the Shannon-Wiener index as cultivation proceeded (Fig 2B). That is, at earlier stages, species numbers in the fermentation systems fell compared to those observed in the fecal inocula, but numbers subsequently rose to levels almost the same as the original ones by 24 h. On the other hand, a PCA indicated a transition in the composition of the microbiota at the species level in fermentation systems (Fig 3). Compositions of the bacterial community in the single-batch fermentation cultures diverged greatly from those of the original fecal samples at earlier fermentation stages, but gradually came back to those of the respective inocula at later time points, coinciding with the tendencies observed in diversity and number of the species. These indicated that most of the microbial species in the original fecal samples were maintained in the fermentation system, although the proportions/numbers of some species changed during the *in vitro* cultivation.

### Short-chain fatty acids in the single-batch fermentation cultures

**Fig 4:**
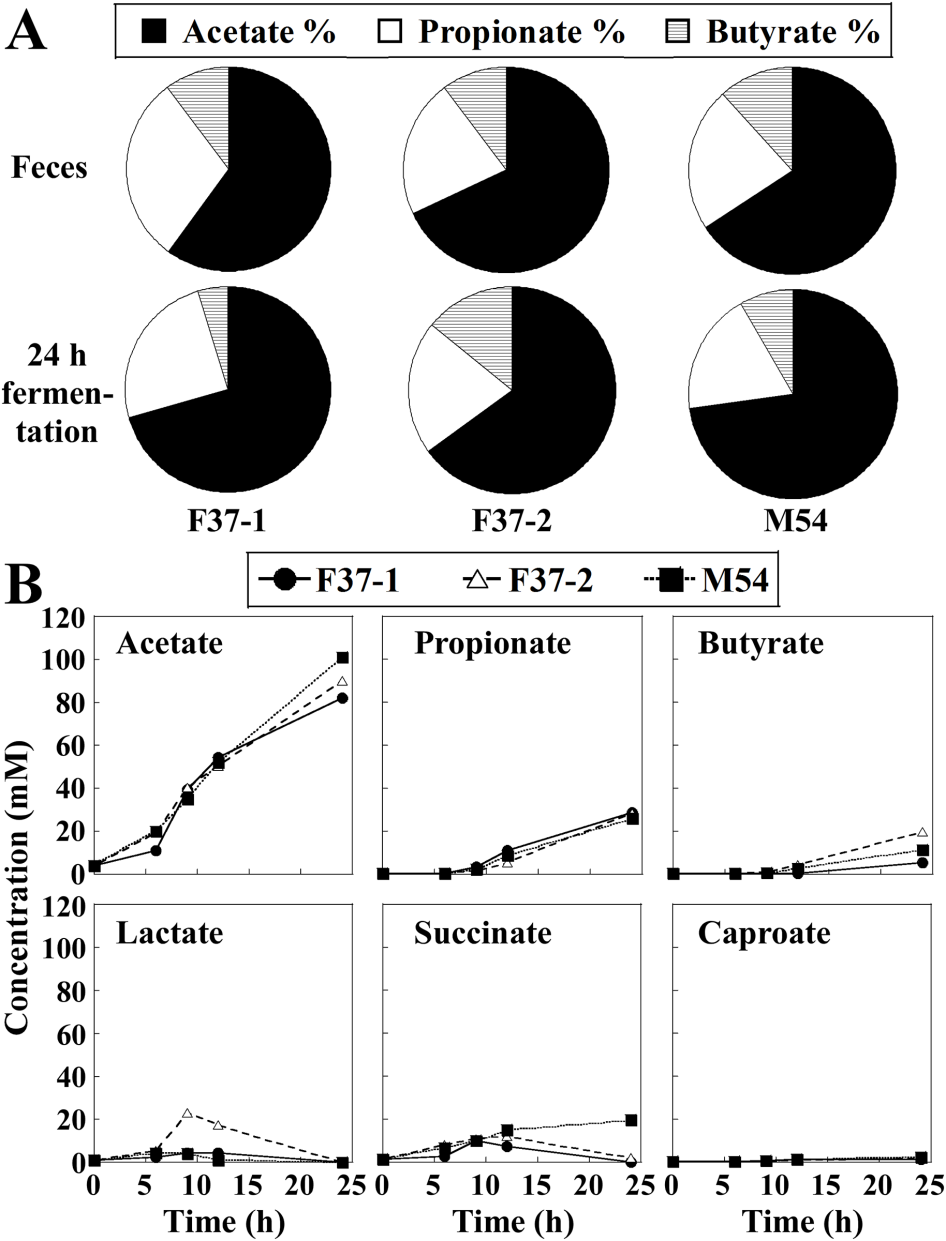
(A) Ratios of acetate, propionate, and butyrate in three human volunteers’ feces (F37-1: female, age 37; F37-2: female, age 37; and M54: male, age 54) and corresponding cultures at 24 h after the initiation of fermentation. (B) Time-dependent change of concentrations of short chain fatty acids (SCFAs) in single-batch fermentation cultures. Values of the ratios are shown as the percentage of the sum of acetate, propionate, and butyrate concentrations.

Short-chain fatty acids (SCFA) are metabolic products of the human gut microbiota that are absorbed by the host; these metabolites have been associated with benefits for host health [38]. Acetate, propionate, and butyrate are the most abundant (≥95%) SCFAs in the human colon and stool, and are present in an approximate molar ratio of 60:20:20 [39]. Therefore, we monitored the SCFA profiles in the *in vitro* fermentation cultures. In the three fecal samples obtained from human volunteers, SCFAs consisted mainly of acetate, propionate, and butyrate (Fig 4A). The single-batch fermentation system showed a constant increase in acetate, propionate, and butyrate throughout the operational duration (Fig 4B). At 24 h after the initiation of fermentation, acetate became the predominant SCFA, followed by propionate and butyrate, almost consistent with the pattern in the original human fecal samples (Fig 4A). Through the first 9 h after the initiation of fermentation, production of lactate and succinate was observed, along with subsequent lactate consumption in F37-1, F37-2, and M54 and succinate consumption in F37-1 and F37-2 cultures (Fig 4B). Caproate was detected only at very low levels throughout the operation.

### Effect of prebiotics on numbers of the genus *Bifidobacterium*

FOS, GOS, IMO, XOS, raffinose, lactulose, and lactosucrose are candidate prebiotic oligosaccharides [38,40,41]. We evaluated the effect on bacteria of genus *Bifidobacterium* of adding each of these prebiotic oligosaccharides to the single-batch fermentation system. For these experiments, inocula were obtained from six human volunteers (F37: female, age 37; F23: female, age 23; M38: male, age 38; F35: female, age 35; M24: male, age 24; M43: male, age 43). At 24 h after the initiation of fermentation, the numbers of total eubacteria and those belonging to genus *Bifidobacterium* were estimated by quantitative PCR analysis.

**Fig 5:**
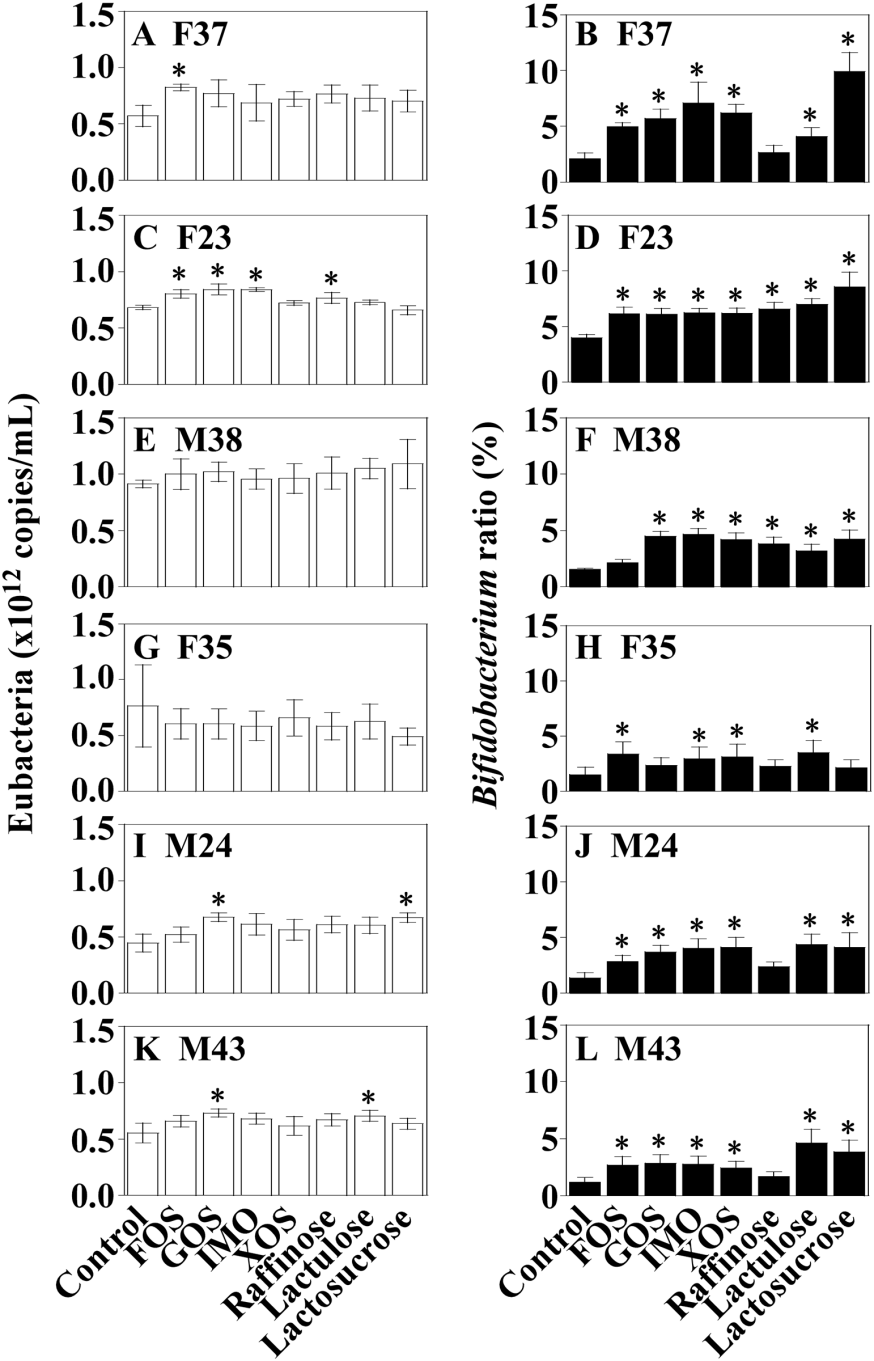
16S rRNA gene copy numbers of eubacteria (A, C, E, G, I, and K) and the ratio of genus *Bifidobacterium* to eubacteria (B, D, F, H, J, and L). Human feces was used to inoculate cultures in the single-batch fermentation system (feces; F37: A and B; F23: C and D; M38: E and F; F35: G and H; M24: I and J; M43: K and L). Samples are labelled according to the prebiotic oligosaccharides [control (no addition), FOS, GOS, IMO, XOS, raffinose, lactulose, and lactosucrose]. Error bars show standard deviation for mean of triplicates. Asterisks indicate values that are significantly different (*p*<0.05) from those for the control systems (n = 3) using the Dunnett test.

In majority of the cases (33 of 42 total trials), eubacterial copy numbers were not significantly changed (compared to the control system) by the addition of prebiotic oligosaccharides to the medium (Fig 5). However, in 35 cases out of 42 total trials, addition of the prebiotic oligosaccharides yielded a significant increase (compared to unsupplemented control cultures) in the ratios of bacteria belonging to genus *Bifidobacterium* as a fraction of total eubacteria (Fig 5). Among the prebiotics, the sole exception was raffinose: in cultures from 4 of 6 human subjects (including F37, F35, M24, and M43 inocula), its addition did not increase the proportion of bacteria of genus *Bifidobacterium*. In addition, raffinose did not increased acetate production at 24 h after the initiation of fermentation, in contrast to significant elevation in acetate production observed with other the prebiotic oligosaccharides (Table 1). On the other hand, increases or decreases in the production of propionate and butyrate were not found in cultures that incorporated any of the prebiotic oligosaccharides.

**Table 1:** Changes in production of acetate, propionate, and butyrate after 24 h of fermentation in the single-batch systems using medium supplemented with the prebiotics (FOS, GOS, IMO, XOS, raffinose, lactulose, or lactosucrose). Samples of human feces (designated F37, F23, M38, F35, M24, or M43) were used to inoculate each system. Changes are presented as the ratio of concentration in the experimental system normalized to that in the control system (without added prebiotics). The control system generated acetate, propionate, and butyrate at concentrations (mean±SD, n = 6) of 104.0±16.3, 28.4±10.1, and 14.8±5.3 mM, respectively.

		Change	
Prebiotics	Acetate	Propionate	Butyrate
FOS	1.18 ± 0.04*	1.02 ± 0.17	1.26 ± 0.19
GOS	1.25 ± 0.06*	1.11 ± 0.22	1.06 ± 0.25
IMO	1.24 ± 0.09*	1.04 ± 0.19	1.06 ± 0.16
XOS	1.23 ± 0.08*	1.11 ± 0.20	1.07 ± 0.14
Raffinose	1.14 ± 0.11	1.14 ± 0.27	1.18 ± 0.18
Lactulose	1.24 ± 0.11*	1.08 ± 0.29	1.16 ± 0.25
Lactosucrose	1.20 ± 0.14*	1.11 ± 0.44	1.19 ± 0.45

* Asterisks indicate values significantly different (*p*<0.05) from the control values (n = 6) using the Dunnett test.

## Discussion

In this study, the total number of eubacteria in the inoculating fecal samples was calculated to range 60.9 × 10^11^ to 128 × 10^11^ copies/wet-g of feces, values comparable to those that have been reported for an average human fecal content (10^12^ microbes/g of feces) [42,43]. The number of eubacteria in the 24 h cultures of our single-batch fermentation system ranged from 4.86 × 10^11^ to 6.93 × 10^11^ copies/mL. Since our batch fermentation system was not equipped with any water-absorbing function such as in the actual intestine, the water content of the culture was approximately ten times as high as that of the feces. In addition, actual dry biomass amount in our batch fermentation system (63.4 g/L) was much higher than that in three-stage bioreactors (2.0–3.7 g/L) [44]. Therefore, the numbers of fecal eubacteria achieved in our single-batch fermentation system were roughly equivalent to those reported in previous studies.

The single-batch fermentation system maintained and simulated the numbers of the bacterial species that predominated in the original fecal samples. The fermentation system achieved bacterial species’ diversities comparable not only to those of the original fecal samples but also to those previously described for human gut microbiota, which were shown to encompass some 400–1,000 species [1,2]. Coinciding with what were reported previously for the human gut microbiota [1,23,42,45], the *in vitro* cultures of our fermentation system were dominated by species belonging to the phyla Bacteroidetes and Firmicutes. Genera *Bacteroides*, *Blautia*, *Ruminococcus*, and *Bifidobacterium* are commonly found in the human gut [5,46], and bacteria belonging to these genera were maintained in the fermentation system. However, bacteria belonging to genera *Escherichia* and *Serratia* dominated at early time points in the fermentation cultures. Prevalence of bacteria of these genera subsequently fell during the 24 h of cultivation, but those belonging to genera *Escherichia* and *Serratia* persisted at much higher numbers *in vitro* than were observed in the inocula. Notably, the increased dominance (at 6 and 9 h after inoculation) of bacteria belonging to genera *Escherichia* and *Serratia* might cause reduced diversity of bacterial species. Viable strains of *E*. *coli* contained in the original fecal samples might grow more rapidly than other bacterial species, given that *E*. *coli* is known as one of the fastest-growing bacterial species exhibiting a doubling time of around 20 min under ideal conditions [47]. We postulate that the lower bacterial diversity observed at the early phase of cultivation was due to the predominance of fast-growing bacteria, although it is likely that relatively slow-growing bacteria might increase their numbers at later phases in cultivation. Previously, the intestinal microbiota of the newborn was characterized to have a lower diversity with relative dominance of bacteria belonging to phyla Proteobacteria and Actinobacteria; thereafter, the microbiota became more diverse with emergence of dominating members belonging to phyla Firmicutes and Bacteroidetes, that are characteristic of the microbiota in adults [48]. This observation imply that dominance of faster-growing species might be a natural cause of decreased diversity in microbiota. To optimize the simulation performance of our *in vitro* system, we would consider controlling the rapidly growing bacteria such as *E*. *coli*. In addition, the decreased prevalence of bacteria belonging to genus *Akkermansia* in our system suggests that some mucin-associated microbes are unable to grow in the current version of our fermentation system. Previous work showed that strains of *Akkermansia muciniphila*, one of the most commonly found members of genus *Akkermansia* in the human intestinal tract, are specialized for degradation and utilization of mucin [49]. Thus, additional conditions and components mimicking the mucosal environment will be incorporated to further optimize our system.

Compositions of SCFAs were similar between the human fecal samples and the cultures of our single-batch fermentation systems at 24 h after the initiation of fermentation. Notably, acetate was the most predominant, followed by propionate and butyrate. Bacteria belonging to phylum Bacteroidetes are known to produce primarily acetate along with propionate, while those of phylum Firmicutes generate butyrate as their primary metabolic end product [39]. Thus, the observed dominance of acetate, propionate, and butyrate coincided with the microbiota analysis, which revealed that bacteria of phyla Bacteroidetes and Firmicutes were the most abundant in both the fecal inocula and the *in vitro* cultures. In the single-batch fermentation system, both lactate and succinate were produced on the earlier stages and readily consumed on later stages. This pattern was comparable to what was reported previously for the actual human intestinal tract, where lactate and succinate were present in the ileum of the small intestine but subsequently depleted in the cecum [50]. Furthermore, at 24 h after the initiation of fermentation, compositions of SCFAs became very similar to those reported for the human large intestine. Based on these results, the temporal shift in metabolic profile (i.e., accumulation/degradation of the metabolic end products) in our simple batch fermentation system appears to mimic the progressive change in the environment in human intestinal tract. Such a time-dependent shift in SCFA production in the fermentation system might be relevant to the changes in proportions and diversity of dominating bacterial species during *in vitro* cultivation.

Prebiotic oligosaccharides are not degraded or absorbed in the stomach and small intestine but are utilized by bacteria of genus *Bifidobacterium* for growth in the human large intestine [41,51]. The bifidogenic properties of the oligosaccharides were observed in our single-batch fermentation system: 6 out of the 7 tested oligosaccharides enhanced the proportion of bacteria of genus *Bifidobacterium* in the complex microbiota at 24 h after the initiation of fermentation. These results were consistent with previous reports [18,52] showing that incorporation into the medium of each of FOS, XOS, GOS, IMO, and lactulose yielded an increase in the number of bacteria of genus *Bifidobacterium* in a batch culture fermenter. Furthermore, the observed increase of acetate production in our system is consistent with the increase in the proportion of bacteria of genus *Bifidobacterium*, given that these bacteria are known to be capable of producing acetate [53,54]. Dinoto and colleagues observed the bifidogenic effect of raffinose in a human feeding trial, in which the members of bifidobacterial species significantly increased in 13 human subjects during a 4-week-long raffinose feeding [55]. In our single-batch fermentation system, however, the bifidogenic effect for raffinose was observed in only 2 cases. These results imply that raffinose might require a longer period of administration in order to exert its bifidogenic effect compared to other oligosaccharides.

## Conclusions

Our single-batch fermentation system supported the growth of bacteria to reach up to the equivalent levels to those seen in the inoculating fecal samples. Bacterial 16S rRNA gene sequence analysis revealed that the system reproduced the microbiota that commonly and dominantly exist in the human colon and successfully maintained the diversity of species found in the original fecal samples. The temporal shift of the metabolic profile in the fermentation system resembled the progressive change in the environment of human intestinal tract. To model mucin layer or mucosal immune system will be our future perspective. Our system at least was able to detect the known bifidogenic effects of prebiotic oligosaccharides, consistent with associated increases in acetate production. Consequently, our single-batch fermentation system is expected to serve as a simple but versatile tool for evaluating the functionality of various food components.

## Supporting Information

S1 Fig (A) Schematic representation and (B) photograph of the single-batch fermentation system.
(TIF)

S2 Fig Genus-level compositional view of bacteria in 3 human fecal samples (–: F37-1, F37-2, and M54) and their corresponding cultures at 6, 9, and 24 h after the initiation of fermentation. Phylum Bacteroidetes includes genera *Bacteroides* and *Parabacteroides*; phylum Firmicutes does genera *Blautia*, *Alkaliphilus*, *Tepidibacter*, *Streptococcus*, *Enterococcus*, *Faecalibacterium*, *Ruminococcus*, *Coprococcus*, and *Megamonas*; phylum Actinobacteria genera *Bifidobacterium* and *Collinsella*; phylum Proteobacteria genera *Escherichia* and *Serratia*; and phylum Verrucomicrobia genus *Akkermansia*. ‘Others’ includes the species belonging to genera whose occupancies were less than 3% of the total numbers.
(TIF)

S1 Table Primers, amplicon sizes, and strains for standard curves used in quantitative PCR detection of target bacteria. (DOCX)

S2 Table Microbiota composition in F37-1 at 24 and 30 h after the initiation of fermentation. (DOCX)
