# Supplementary material for: A Single-Batch Fermentation System to Simulate Human Colonic Microbiota for High-Throughput Evaluation of Prebiotics
Source: PLoS One. 2016 Aug 2;11(8):e0160533. doi: 10.1371/journal.pone.0160533 (PMC4970706; doi:10.1371/journal.pone.0160533)
Supplement: S1 Table — (DOCX) [file pone.0160533.s003.docx]

**S1 Table. Primers, amplicon sizes, and strains for standard curves used in quantitative PCR detection of target bacteria.**

| Target bacteria | Sequences (5' to 3') | Product size (bp) | Annealing temp  (°C) | Strains for standard curves | Ref. |
| --- | --- | --- | --- | --- | --- |
| All eubacteria | F: ACTCCTACGGGAGGCAGCAGT | 200 | 54 | *Escherichia coli* ATCC 43888^T^ | 31 |
|  | R: GTATTACCGCGGCTGCTGGCAC |  |  |  |  |
| *Bifidobacterium* | F: CTCCTGGAAACGGGTGG | 549–563 | 55 | *Biﬁdobacterium catenulatum* JCM 1194^T^ | 32 |
|  | R: GGTGTTCTTCCCGATATCTACA |  |  |  |  |
| *Bacteroides*-*Prevotella*-*Porphyromonas* | F: GGTGTCGGCTTAAGTGCCAT | 140 | 68 | *Bacteroides ovatus* JCM 5824^T^ | 33 |
|  | R: CGGA(C/T)GTAAGGGCCGTGC |  |  |  |  |
| *Clostridium coccoides*-*Eubacterium rectale* group | F: CGGTACCTGACTAAGAAGC | 429 | 55 | *Clostridium symbiosum* JCM 1297^T^ | 33 |
|  | R: AGTTT(C/T)ATTCTTGCGAACG |  |  |  |  |
| *Lactobacillus* group | F: AGCAGTAGGGAATCTTCCA | 341 | 58 | *Lactobacillus plantarum* 22A-3 | 33 |
|  | R: CACCGCTACACATGGAG |  |  |  |  |
| *Enterococcus* spp. | F: CCCTTATTGTTAGTTGCCATCATT | 144 | 61 | *Enterococcus casseliflavus* JCM 8723^T^ | 33 |
|  | R: ACTCGTTGTACTTCCCATTGT |  |  |  |  |
| *Enterobacteriacea*e | F: CATTGACGTTACCCGCAGAAGAAGC | 195 | 63 | *Escherichia coli* ATC 43888 | 34 |
|  | R: CTCTACGAGACTCAAGCTTGC |  |  |  |  |

1. Ahmed S, Macfarlane GT, Fite A, McBain AJ, Gilbert P, Macfarlane S. Mucosa-Associated Bacterial Diversity in Relation to Human Terminal Ileum and Colonic Biopsy Samples. Appl Environ Microbiol. 2007; 73:7435–7442. doi: 10.1128/AEM.01143-07
2. [Matsuki T](http://www.ncbi.nlm.nih.gov/pubmed/?term=Matsuki%20T%5BAuthor%5D&cauthor=true&cauthor_uid=14711639), [Watanabe K](http://www.ncbi.nlm.nih.gov/pubmed/?term=Watanabe%20K%5BAuthor%5D&cauthor=true&cauthor_uid=14711639), [Fujimoto J](http://www.ncbi.nlm.nih.gov/pubmed/?term=Fujimoto%20J%5BAuthor%5D&cauthor=true&cauthor_uid=14711639), [Kado Y](http://www.ncbi.nlm.nih.gov/pubmed/?term=Kado%20Y%5BAuthor%5D&cauthor=true&cauthor_uid=14711639), [Takada T](http://www.ncbi.nlm.nih.gov/pubmed/?term=Takada%20T%5BAuthor%5D&cauthor=true&cauthor_uid=14711639), [Matsumoto K](http://www.ncbi.nlm.nih.gov/pubmed/?term=Matsumoto%20K%5BAuthor%5D&cauthor=true&cauthor_uid=14711639), et al. Quantitative PCR with 16S rRNA-gene-targeted species-specific primers for analysis of human intestinal bifidobacteria. Appl Environ Microbiol. 2004; 70:167–173. doi: 10.1128/AEM.70.1.167-173.2004
3. Rinttilä T, Kassinen A, Malinen E, Krogius L, Palva A. Development of an extensive set of 16S rDNA-targeted primers for quantification of pathogenic and indigenous bacteria in faecal samples by real-time PCR. J Appl Microbiol. 2004; 97:1166‒1177. doi: [10.1111/j.1365-2672.2004.02409.x](http://dx.doi.org/10.1111/j.1365-2672.2004.02409.x)
4. Bartosch S, Fite A, Macfarlane GT, McMurdo MET. Characterization of bacterial communities in feces from healthy elderly volunteers and hospitalized elderly patients by using real-time PCR and effects of antibiotic treatment on the fecal microbiota. Appl Environ Microbiol. 2004; 70:3575‒3581. doi: [10.1128/AEM.70.6.3575-3581.2004](http://dx.doi.org/10.1128/AEM.70.6.3575-3581.2004)
