# Supplementary material for: A Single-Batch Fermentation System to Simulate Human Colonic Microbiota for High-Throughput Evaluation of Prebiotics
Source: PLoS One. 2016 Aug 2;11(8):e0160533. doi: 10.1371/journal.pone.0160533 (PMC4970706; doi:10.1371/journal.pone.0160533)
Supplement: S2 Table — (DOCX) [file pone.0160533.s004.docx]

**S2 Table. Microbiota composition in F37-1 at 24 and 30 h after the initiation of fermentation.**

| Time | *Bacteroides*-*Prevotella*-*Porphyromonas* | *Clostridium coccoides*-*Eubacterium rectale* group | *Lactobacillus* group | *Enterococcus* spp. | *Enterobacteriaceae* |
| --- | --- | --- | --- | --- | --- |
|  | (×10^6^ copies per mL) | (×10^6^ copies per mL) | (×10^6^ copies per mL) | (×10^6^ copies per mL) | (×10^6^ copies per mL) |
| 24 h | 190 | 4900 | 2.0 | 770 | 1600 |
| 30 h | 160 | 4200 | 3.1 | 570 | 1300 |
